# Comparison of the Effectiveness of Baloxavir and Oseltamivir in Outpatients With Influenza B

**DOI:** 10.1111/irv.70002

**Published:** 2024-08-27

**Authors:** Takahiro Takazono, Genta Ito, Naoki Hosogaya, Naoki Iwanaga, Takuji Komeda, Masayuki Kobayashi, Yoshitake Kitanishi, Eriko Ogura, Hiroshi Mukae

**Affiliations:** ^1^ Department of Respiratory Medicine Nagasaki University Hospital Nagasaki Japan; ^2^ Department of Infectious Diseases Nagasaki University Graduate School of Biomedical Sciences Nagasaki Japan; ^3^ Data Science Department, Shionogi & Co., Ltd. Osaka Japan; ^4^ Clinical Research Center Nagasaki University Hospital Nagasaki Japan; ^5^ Global Development Division, Shionogi & Co., Ltd. Tokyo Japan; ^6^ Department of Respiratory Medicine Nagasaki University Graduate School of Biomedical Sciences Nagasaki Japan

**Keywords:** baloxavir, database, hospitalization, influenza, Japanese, oseltamivir

## Abstract

This retrospective cohort study analyzed data from a Japanese health insurance database to assess the effectiveness of baloxavir (*n* = 4822) for preventing severe events compared with oseltamivir (*n* = 10,523) in patients with influenza B. The primary endpoint was hospitalization incidence (Days 2–14). The secondary endpoints included intravenous antibacterial drug use, pneumonia hospitalization, heart failure hospitalization, inhalational oxygen requirement, and use of other anti‐influenza drugs. The hospitalization incidence was significantly lower with baloxavir (0.15% vs. 0.37%; risk ratio: 2.48, 95% confidence interval: 1.13–5.43). Pneumonia and additional anti‐influenza therapy were also less frequent with baloxavir, thus supporting its use.

**Trial Registration:** UMIN Clinical Trials Registry Study ID: UMIN000051382

The influenza B virus accounts for approximately 23% of influenza infections each season [1]. In Japan, four neuraminidase inhibitors are available for influenza treatment: oseltamivir phosphate (oseltamivir), zanamivir, laninamivir, and peramivir [2]. Baloxavir marboxil (baloxavir) is a cap‐dependent endonuclease inhibitor with a novel mechanism of action that was approved for influenza treatment in Japan in 2018 [3] and is now used in other parts of the world [4]. A previous database study found that baloxavir may be associated with a lower incidence of hospitalization than oseltamivir in patients with seasonal influenza [5]. However, these findings were considered primarily in the context of influenza A, which accounted for approximately 70% of infections in the study population. Influenza B accounted for only around 1% of infections, and the influenza type was unknown in the remaining 29% of cases. Another clinical trial reported that patients with an influenza B infection and treated with baloxavir had a significantly shorter duration of symptom than those with oseltamivir [6]. However, this trial focused on patients at high risk of influenza‐related complications.

To our knowledge, no studies have reported the effectiveness of baloxavir for preventing severe events in patients with influenza B. Therefore, we conducted this retrospective database cohort study to compare the incidence of severe events, including hospitalization, between patients treated with baloxavir and those treated with oseltamivir for influenza B during the 2018/2019 and 2019/2020 influenza seasons.

We utilized a Japanese database (JMDC; JMDC Inc., Tokyo, Japan) containing inpatient, outpatient, and prescription receipts from health insurance associations. Patient data from April 1, 2018, to April 30, 2020, were extracted to cover the 2018/2019 and 2019/2020 Japanese influenza seasons. Patients who were diagnosed with influenza B during the 2018/2019 (October 1, 2018–April 17, 2019) or 2019/2020 (October 1, 2019–April 17, 2020) Japanese influenza seasons, registered in the JMDC database for ≥ 6 months prior to the diagnosis date (Day 1), and prescribed baloxavir or oseltamivir on Day 1 were eligible. No specific method for influenza B diagnosis was identified in the data; therefore, the availability of information on the diagnostic method was not considered an eligibility criterion in the present study. Patients who were aged < 1 year on Day 1, prescribed antibacterial agents (European Pharmaceutical Market Research Association Anatomical Therapeutic Chemical code J01) on Day 1, diagnosed with pneumonia (International Statistical Classification of Diseases and Related Health Problems, 10th Revision codes J12–J18) on Day 1, prescribed multiple anti‐influenza drugs (including baloxavir, oseltamivir, zanamivir, laninamivir, and peramivir) on Day 1, hospitalized on Day 1, or died on Day 1 were excluded. The background data collected are described in the Supporting Information.

This study was conducted according to the Ethical Guidelines for Medical and Health Research Involving Human Subjects. As this study utilized anonymized information from an existing database, informed consent was not required. The primary endpoint was the hospitalization incidence between Days 2 and 14. The secondary endpoints were the incidences of intravenous antibacterial drug use, pneumonia hospitalization, use of intravenous antibacterial drugs for pneumonia, inhaled oxygen requirement for pneumonia, use of anti‐influenza drugs other than the drug prescribed on Day 1, and heart failure hospitalization between Days 2 and 14. Meningitis was an exploratory endpoint. Additional details are included in the Supporting Information.

Propensity scores were calculated by logistic regression with the baloxavir group as the objective variable and patient baseline characteristics as explanatory variables. Treatment groups were balanced using the inverse probability of treatment weighting (IPTW) method. Characteristics related to influenza severity were used as covariates and are listed in the Supporting Information. Between‐group standardized mean differences (SMDs) were calculated for each baseline characteristic. Missing data were not imputed, and multiplicity was not adjusted for repeated tests. The incidence of each endpoint in the oseltamivir group was divided by the incidence in the baloxavir group to calculate the risk ratio (RR). The incidence in the oseltamivir group was subtracted from the incidence in the baloxavir group to calculate risk differences (RDs), and 95% confidence intervals (CIs) were calculated for both RRs and RDs. The groups were considered different when the 95% CIs for the RRs and RDs did not span the values 1.00 or 0.00, respectively. Statistical analyses had a two‐sided significance level of 5% and were conducted using SAS version 9.4 (SAS Institute Inc., Cary, NC, US).

During the 2018/2019 and 2019/2020 influenza seasons in Japan, 16,507 eligible patients were identified in the JMDC claims database (Figure 1). Among 15,345 patients analyzed, 4822 (31.4%) and 10,523 (68.6%) received baloxavir and oseltamivir, respectively. Fewer patients were aged < 5 years in the baloxavir group than in the oseltamivir group (1.7% vs. 20.6%, respectively) (Table 1). After IPTW adjustment, the SMDs between the baloxavir and oseltamivir groups were < 0.1 for all characteristics, indicating that the baseline characteristics were similar after adjustment.

**FIGURE 1:**
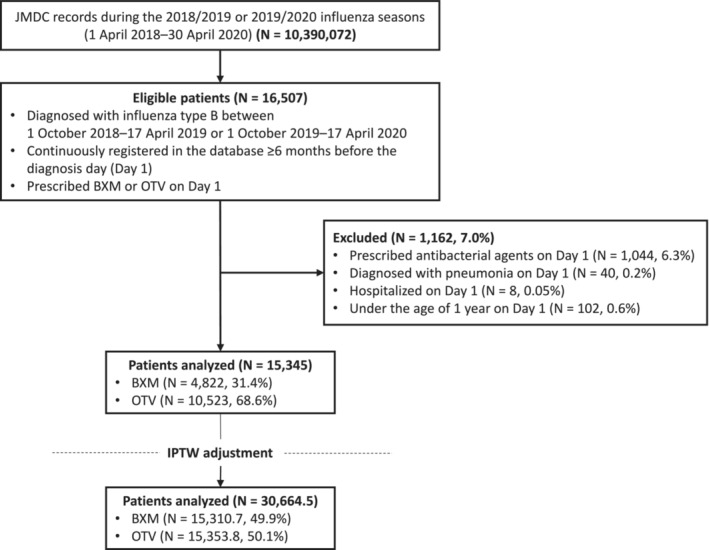
Study flow diagram. Abbreviations: BXM, baloxavir marboxil; OTV, oseltamivir phosphate.

**TABLE 1:** Patient baseline characteristics.

	Unadjusted		Adjusted by IPTW method		SMD
	BXM (*n* = 4822)	OTV (*n* = 10,523)	BXM (*n* = 15,310.7)	OTV (*n* = 15,353.8)	
Age, years					
1 to < 2	5 (0.1)	294 (2.8)	306.4 (2.0)	299.0 (1.9)	< 0.01
≥ 2 to < 5	76 (1.6)	1870 (17.8)	1936.8 (12.7)	1946.0 (12.7)	< 0.01
≥ 5 to < 18	2388 (49.5)	6082 (57.8)	8419.1 (55.0)	8465.4 (55.1)	< 0.01
≥ 18 to < 65	2337 (48.5)	2264 (21.5)	4620.0 (30.2)	4615.3 (30.1)	< 0.01
≥ 65	16 (0.3)	13 (0.1)	28.4 (0.2)	28.1 (0.2)	< 0.01
Sex					
Female	2060 (42.7)	4891 (46.5)	6886.9 (45.0)	6951.0 (45.3)	< 0.01
Male	2762 (57.3)	5632 (53.5)	8423.8 (55.0)	8402.8 (54.7)	< 0.01
Steroid use	21 (0.4)	33 (0.3)	59.1 (0.4)	56.3 (0.4)	< 0.01
Antibacterial use	1246 (25.8)	3059 (29.1)	4319.3 (28.2)	4306.9 (28.1)	< 0.01
Previous hospitalization	27 (0.6)	109 (1.0)	92.9 (0.6)	134.1 (0.9)	0.03
Comorbidities					
Pneumonia	29 (0.6)	109 (1.0)	148.6 (1.0)	138.8 (0.9)	< 0.01
Respiratory infection other than pneumonia	3155 (65.4)	6805 (64.7)	9865.6 (64.4)	9939.5 (64.7)	< 0.01
Asthma	999 (20.7)	3800 (36.1)	4762.7 (31.1)	4775.0 (31.1)	< 0.01
Diabetes mellitus	34 (0.7)	34 (0.3)	67.1 (0.4)	68.8 (0.4)	< 0.01
Chronic obstructive pulmonary disease	31 (0.6)	55 (0.5)	88.7 (0.6)	85.1 (0.6)	< 0.01
Cardiovascular disease	31 (0.6)	92 (0.9)	144.8 (0.9)	123.0 (0.8)	0.02
Cerebrovascular disease	27 (0.6)	27 (0.3)	53.7 (0.4)	54.6 (0.4)	< 0.01
Psychiatric disease, including dementia	260 (5.4)	690 (6.6)	854.9 (5.6)	945.0 (6.2)	0.02
Neurological disease	267 (5.5)	379 (3.6)	639.3 (4.2)	656.8 (4.3)	< 0.01
Anemia	82 (1.7)	141 (1.3)	255.7 (1.7)	226.4 (1.5)	0.02
Immune deficiency	5 (0.1)	10 (0.1)	11.8 (0.1)	14.1 (0.1)	< 0.01
Moderate or severe liver disease	1 (0.0)	1 (0.0)	1.5 (0.0)	1.5 (0.0)	< 0.01
Malignant tumor	24 (0.5)	37 (0.4)	65.5 (0.4)	61.8 (0.4)	< 0.01

*Note:* Data are shown as *n* (%).

Abbreviations: BXM, baloxavir marboxil; IPTW, inverse probability of treatment weighting; OTV, oseltamivir phosphate; SMD, standardized mean difference.

After IPTW adjustment, the hospitalization incidence was significantly lower in the baloxavir group than in the oseltamivir group (0.15% vs. 0.37%, respectively; RR: 2.48, 95% CI: 1.13–5.43; RD: 0.22, 95% CI: 0.06–0.38) (Figure 2). For the post hoc sensitivity analysis, data for patients aged < 5 years were removed because of age distribution bias, as fewer patients in the baloxavir group were aged < 5 years. The hospitalization incidence remained lower in the baloxavir group; however, the difference was no longer statistically significant (0.18% vs. 0.35%; RR: 1.98, 95% CI: 0.89–4.43; RD: 0.17, 95% CI: −0.01 to 0.35) (Figure S1). The incidences of pneumonia (0.16% vs. 0.57%) and additional anti‐influenza therapy use (0.22% vs. 0.57%) were significantly lower with baloxavir than with oseltamivir (Figure 2), with an RR for pneumonia of 3.59 (95% CI: 1.84–6.98; RD: 0.41, 95% CI: 0.24–0.59) and an RR for additional anti‐influenza therapy use of 2.57 (95% CI: 1.31–5.03; RD: 0.35, 95% CI: 0.15–0.55).

**FIGURE 2:**
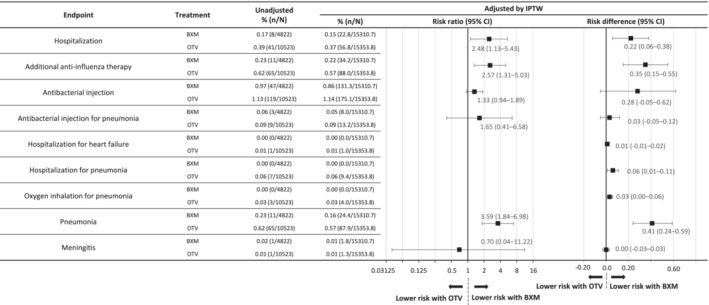
Incidence and risk of events in patients with influenza B virus treated with BXM or OTV. The incidence of each endpoint in the oseltamivir group was divided by the incidence in the baloxavir group to calculate the RR. The incidence in the oseltamivir group was subtracted from the incidence in the baloxavir group to calculate RDs, and 95% CIs were calculated for both RRs and RDs. The groups were considered different when the 95% CIs for the RRs and RDs did not span the values 1.00 or 0.00, respectively. The RR is presented on a log base 2 scale. Abbreviations: BXM, baloxavir marboxil; CI, confidence interval; IPTW, inverse probability of treatment weighting; OTV, oseltamivir phosphate; RD, risk difference; RR, risk ratio.

This is the first real‐world report to examine whether baloxavir reduces influenza B infection severity in the outpatient setting. Patients with influenza B treated with baloxavir had a lower hospitalization incidence than those treated with oseltamivir. Additionally, the baloxavir group had lower incidences of pneumonia and additional anti‐influenza therapy use than the oseltamivir group.

The design of this study and the previous database study were similar, with some key differences [5]. We included patients who were hospitalized, treated with antimicrobials, or diagnosed with pneumonia in the 90 days before Day 1 to ensure those at high risk of pneumonia were included whereas the previous study did not [5]. Moreover, we only included patients with influenza B. Additionally, we did not include use of oral antimicrobials as a secondary endpoint, as these are sometimes inappropriately used in Japan [7]. The previous database study, in which 70% of patients had influenza A, found a smaller hospitalization RR and RD between baloxavir and oseltamivir (RR: 1.41, 95% CI: 1.00–2.00; RD: 0.06, 95% CI: 0.01–0.12) [5], suggesting that baloxavir may have greater effectiveness at treating influenza B and preventing hospitalization.

Baloxavir treatment of influenza B is associated with shorter fever and disease duration than oseltamivir treatment [8]. Oseltamivir has a higher 50% inhibitory concentration against influenza B than against influenza A. Additionally, baloxavir has faster yield reduction results than oseltamivir [9], indicating that oseltamivir may be less potent for treating influenza B. Oseltamivir is also reported to have lower clinical effectiveness in infants and children with influenza B [10]. Overall, our findings are consistent with previous studies and provide evidence that baloxavir may be more effective at preventing severe events in patients with influenza B than oseltamivir.

Although 28.9% of Japan's population is aged ≥ 65 years [11], only 1% of the JMDC database represents older patients [12]; thus, our findings may not be generalizable to elderly or pediatric groups. As the database relies on receipt information, it lacks detailed clinical information. Therefore, influenza severity at treatment initiation and vaccination status were unknown. The accuracy and severity of the covariates were uncertain, including pneumonia diagnosis.

In conclusion, this real‐world database study found that patients with influenza B treated with baloxavir had a reduced incidence of severe events, including hospitalization, pneumonia, and use of additional anti‐influenza therapies, than those treated with oseltamivir.

## Author Contributions

**Takahiro Takazono:** conceptualization, methodology, project administration, writing–review and editing, writing – original draft. **Genta Ito:** formal analysis, investigation, writing – review and editing, writing – original draft, methodology, validation. **Naoki Hosogaya:** conceptualization, methodology, project administration, writing – review and editing, writing – original draft. **Naoki Iwanaga:** writing – review and editing, writing – original draft. **Takuji Komeda:** conceptualization, formal analysis, investigation, methodology, validation, writing – review and editing, writing – original draft. **Masayuki Kobayashi:** formal analysis, writing – review and editing, writing–original draft. **Yoshitake Kitanishi:** funding acquisition, writing – original draft, writing – review and editing, supervision. **Eriko Ogura:** conceptualization, writing–original draft, writing – review and editing, methodology. **Hiroshi Mukae:** funding acquisition, writing – original draft, writing – review and editing, supervision.

## Conflicts of Interest

Genta Ito, Takuji Komeda, Masayuki Kobayashi, Yoshitake Kitanishi, and Eriko Ogura are employees of Shionogi & Co., Ltd. Yoshitake Kitanishi holds stocks in Shionogi & Co., Ltd. Takahiro Takazono has received personal fees from Shionogi & Co., Ltd., MSD Co., Ltd., Pfizer Inc., Insmed Japan, Asahi Kasei Pharma Corporation, and Kyorin Pharmaceutical Co., Ltd. Hiroshi Mukae has also received personal fees from AbbVie GK., Asahi Kasei Pharma Corporation, Astellas Pharma Inc., AstraZeneca K.K., Bristol‐Myers Squibb, Eli Lilly Japan K.K., FUJIFILM Toyama Chemical Co., Ltd., Gilead Sciences Inc., Insmed Incorporated, Janssen Pharmaceutical K.K., Kyorin Pharmaceutical Co., Ltd., Meiji Seika Pharma Co., Ltd., Mitsubishi Tanabe Pharma Corporation, MSD Co., Ltd., Nihon Pharmaceutical Co., Ltd., Nippon Boehringer Ingelheim Co., Ltd., Novartis Pharma K.K., Pfizer Inc., Sumitomo Dainippon Pharma Co., Ltd., Taiho Pharmaceutical Co., Ltd., Taisho Pharma Co., Ltd., Teijin Home Healthcare Ltd., and Toa Shinyaku Co., Ltd. and grants from Asahi Kasei Pharma Corporation, Astellas Pharma Inc., FUJIFILM Toyama Chemical Co., Ltd., Kyorin Pharmaceutical Co., Ltd., Meiji Seika Pharma Co., Ltd., Pfizer Inc., Taiho Pharmaceutical Co., Ltd., Taisho Pharma Co., Ltd., Teijin Pharma Ltd., Toa Shinyaku Co., Ltd., and Torii Pharmaceutical Co., Ltd., outside the submitted work. Naoki Hosogaya and Naoki Iwanaga have no conflicts of interest to disclose.

## Supporting information


**Figure S1** Sensitivity analysis of the incidence and risk of events in patients aged ≥& 5 years with influenza B treated with BXM or OTV.

**Data S1** Supporting Information.

## Data Availability

The data that support the findings of this study are available from JMDC Inc. Restrictions apply on the availability of these data, which were used under license for this study. Data are available from the authors with the permission of JMDC Inc.
