# Supplementary material for: Comparison of the Effectiveness of Baloxavir and Oseltamivir in Outpatients With Influenza B
Source: Influenza Other Respir Viruses. 2024 Aug 27;18(9):e70002. doi: 10.1111/irv.70002 (PMC11347862; doi:10.1111/irv.70002)
Supplement: Supplementary file 1 — Figure S1 Sensitivity analysis of the incidence and risk of events in patients aged ≥& 5 years with influenza B treated with BXM or OTV. [file IRV-18-e70002-s001.pptx]

## Slide 1
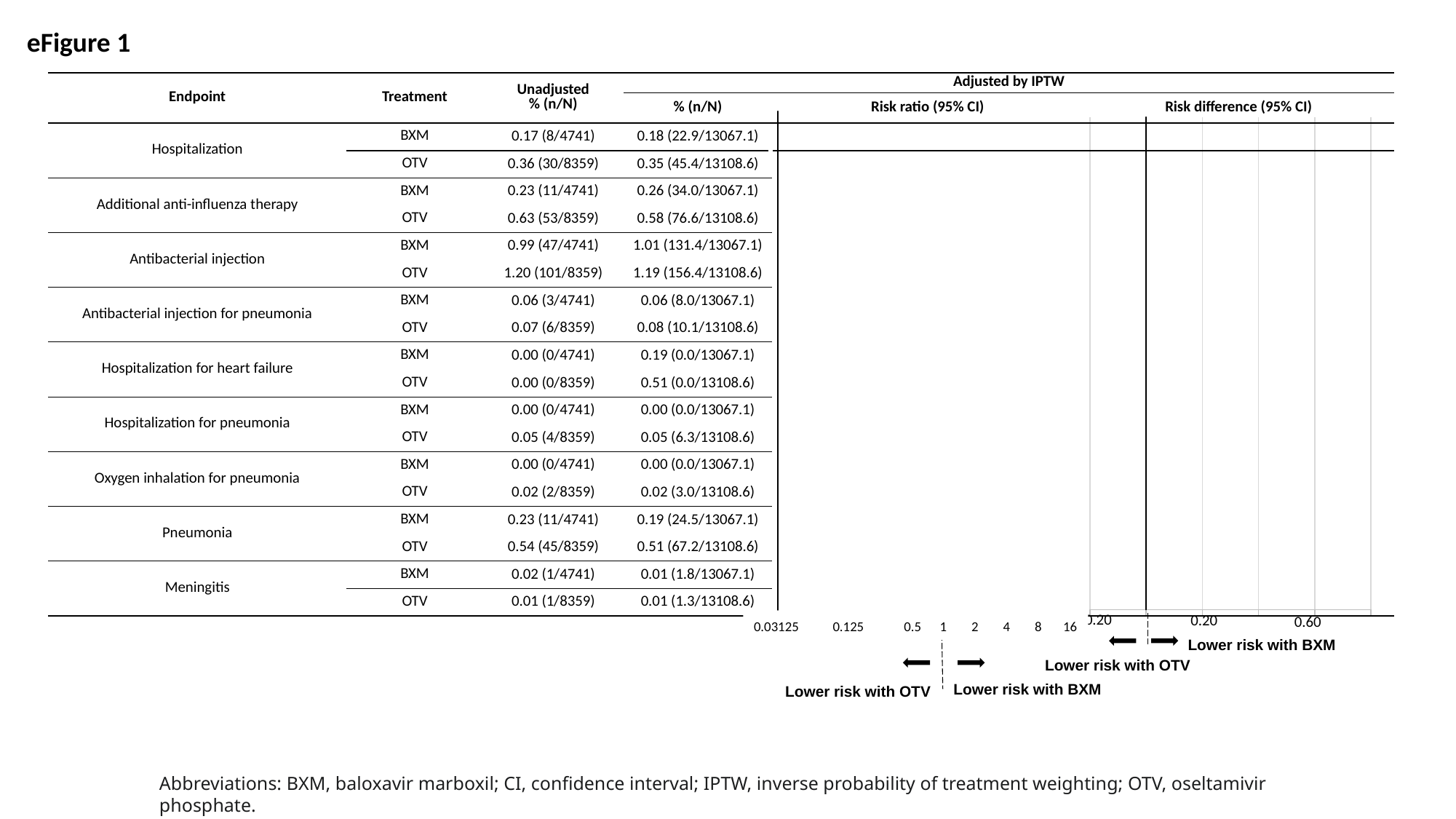

eFigure 1
| Endpoint | Treatment | Unadjusted % (n/N) | Adjusted by IPTW | | |
| --- | --- | --- | --- | --- | --- |
| | | | % (n/N) | Risk ratio (95% CI) | Risk difference (95% CI) |
| Hospitalization | BXM | 0.17 (8/4741) | 0.18 (22.9/13067.1) | | |
| | OTV | 0.36 (30/8359) | 0.35 (45.4/13108.6) | | |
| Additional anti-influenza therapy | BXM | 0.23 (11/4741) | 0.26 (34.0/13067.1) | | |
| | OTV | 0.63 (53/8359) | 0.58 (76.6/13108.6) | | |
| Antibacterial injection | BXM | 0.99 (47/4741) | 1.01 (131.4/13067.1) | | |
| | OTV | 1.20 (101/8359) | 1.19 (156.4/13108.6) | | |
| Antibacterial injection for pneumonia | BXM | 0.06 (3/4741) | 0.06 (8.0/13067.1) | | |
| | OTV | 0.07 (6/8359) | 0.08 (10.1/13108.6) | | |
| Hospitalization for heart failure | BXM | 0.00 (0/4741) | 0.19 (0.0/13067.1) | | |
| | OTV | 0.00 (0/8359) | 0.51 (0.0/13108.6) | | |
| Hospitalization for pneumonia | BXM | 0.00 (0/4741) | 0.00 (0.0/13067.1) | | |
| | OTV | 0.05 (4/8359) | 0.05 (6.3/13108.6) | | |
| Oxygen inhalation for pneumonia | BXM | 0.00 (0/4741) | 0.00 (0.0/13067.1) | | |
| | OTV | 0.02 (2/8359) | 0.02 (3.0/13108.6) | | |
| Pneumonia | BXM | 0.23 (11/4741) | 0.19 (24.5/13067.1) | | |
| | OTV | 0.54 (45/8359) | 0.51 (67.2/13108.6) | | |
| Meningitis | BXM | 0.02 (1/4741) | 0.01 (1.8/13067.1) | | |
| | OTV | 0.01 (1/8359) | 0.01 (1.3/13108.6) | | |
### Chart
| Category | | |
|---|---|---|
### Chart
| Category | | |
|---|---|---|-0.20
0.20
0.60
0.03125 0.125 0.5 1 2 4 8 16
Lower risk with BXM
Lower risk with OTV
Lower risk with BXM
Lower risk with OTV
Abbreviations: BXM, baloxavir marboxil; CI, confidence interval; IPTW, inverse probability of treatment weighting; OTV, oseltamivir phosphate.
