# Supplementary material for: Comparison of the Effectiveness of Baloxavir and Oseltamivir in Outpatients With Influenza B
Source: Influenza Other Respir Viruses. 2024 Aug 27;18(9):e70002. doi: 10.1111/irv.70002 (PMC11347862; doi:10.1111/irv.70002)
Supplement: Supplementary file 2 — Data S1 Supporting Information. [file IRV-18-e70002-s002.docx]

# Supplementary Material

**Supplementary Methods**

*Patient background data*

The following patient background data were collected: age, sex, steroid use (World Health Organization [WHO] Anatomical Therapeutic Chemical [ATC] code H02), dialysis use (presence of a dialysis-related medical care activity), antibacterial use (European Pharmaceutical Market Research Association [EphMRA]-ATC code J01), history of hospitalization, and presence of the following comorbidities: pneumonia (ICD-10 codes J12–J18), respiratory co-infection other than pneumonia (International Statistical Classification of Diseases and Related Health Problems, 10th Revision [ICD-10] codes J00–006, J2), asthma (WHO-ACT code R03), diabetes mellitus (WHO-ATC code A10), chronic obstructive pulmonary disease (ICD-10 codes J41–J44), cardiovascular disease (ICD-10 codes I20–I25, Q20–Q28), cerebrovascular disease (ICD-10 codes I60–I69), psychiatric disease (including dementia) (ICD-10 codes F00–F99), neurological disease (ICD-10 codes G00–G99), anemia (ICD-10 codes D50–D64), immunodeficiency (ICD-10 codes D80–D89), moderate or severe liver disease (ICD-10 codes I85.0, I85.9, I86.4, I98.2, K70.4, K71.1, K72.1, K72.9, K76.5, K76.6, and K76.7), and malignant tumors (ICD-10 codes D00–D09, C00–C97). The presence of comorbidities was determined when the corresponding ICD-10 code was recorded ≤6 months prior to day 1 (excluding day 1). Comorbidities were also identified by the presence of disease-related medication prescriptions.

*Study outcomes*

The secondary endpoints, intravenous antibacterial drug use and presence of meningitis, were determined by EphMRA-ATC code J01 and ICD-10 codes G00−G03, respectively.

*Covariates*

Covariates included age, sex, and previous medical history (steroid use, dialysis, respiratory infection, diabetes mellitus, chronic obstructive pulmonary disease, cardiovascular disease, cerebrovascular disease, psychiatric disorder including dementia, neurological disease, anemia, immunodeficiency, severe liver disease, malignancy, hospitalization, pneumonia, and antibacterial use).

**Supplementary Figures**

**Supplementary Figure 1. Sensitivity analysis of the incidence and risk of events in patients aged ≥5 years with influenza B treated with BXM or OTV.**

Abbreviations: BXM, baloxavir marboxil; CI, confidence interval; IPTW, inverse probability of treatment weighting; OTV, oseltamivir phosphate.
